# Effects of Nitrogen Addition on Soil Carbon-Fixing Microbial Diversity on Different Slopes in a Degraded Alpine Meadow

**DOI:** 10.3389/fpls.2022.921278

**Published:** 2022-06-24

**Authors:** Chengyi Li, Xilai Li, Yan Shi, Yuanwu Yang, Honglin Li

**Affiliations:** ^1^College of Agriculture and Animal Husbandry, Qinghai University, Xining, China; ^2^State Key Laboratory of Plateau Ecology and Agriculture, Qinghai University, Xining, China; ^3^School of Environment, The University of Auckland, Auckland, New Zealand

**Keywords:** Qinghai–Tibet plateau, nitrogen addition, carbon-fixing bacteria, *cbbL* gene, alpine meadow, stoichiometric ratio

## Abstract

Autotrophic carbon-fixing bacteria are a major driver of carbon sequestration and elemental cycling in grassland ecosystems. The characteristics of the response of carbon-fixing bacterial communities to nitrogen (N) addition in degraded alpine meadows are unclear. In this study, it was investigated that the effects of N addition in three levels [they are low (LN), middle (MN), and high (HN) with N supplement of 2, 5, and 10 g N⋅m^–2^⋅a^–1^, respectively] on soil carbon-fixing bacteria on different slopes in a degraded alpine meadow in the Yellow River on the Qinghai–Tibet Plateau. The results showed that there were significant differences in the abundance of some low abundance genera of carbon-fixing bacteria on the same slope (*P* < 0.05), but the differences in the abundance of various phyla and dominant genera were not significant. MN on gentle slopes significantly reduced the Chao1 index and observed species (*P* < 0.05), whereas N addition on steep slopes had no significant effect on the diversity. The abundance of the Cyanobacteria phylum and 28 genera of identified carbon-fixing bacteria differed significantly between slopes (*P* < 0.05), and observed species of carbon-fixing bacteria were significantly higher on steep slopes than on gentle slopes (*P* < 0.05). Factors affecting the carbon-fixing bacteria community structure include slope, N addition, ammoniacal nitrogen (N-NH_4_^+^), microbial biomass carbon (MBC), soil water content (SWC), pH, soil C:N ratio, and microbial C:N ratio. Slope, N addition, soil physicochemical properties, microbial biomass, and stoichiometric ratio did not significantly affect the carbon-fixing bacteria diversity. Thus, the effect of exogenous N addition on carbon-fixing bacteria in degraded alpine meadows was dependent on slope conditions, and the response of carbon-fixing bacteria abundance and species number to N addition on gently slope sites was threshold-limited.

## Introduction

The global soil carbon pool is about 2.2–3.0 × 10^3^ Pg (Cao et al., 2017), and it is the most important carbon sink for terrestrial ecosystems. The carbon sequestration is three times higher than the terrestrial vegetation carbon pool and four times higher than the atmospheric carbon pool (McCarl et al., 2007; Lacis et al., 2010; Vicente-Vicente et al., 2016). Soil carbon pools occupy an important position in the terrestrial carbon cycle, and small changes in them can lead to an increase in CO_2_ emissions to the atmosphere, altering the global carbon balance and affecting global climate change (Yousuf et al., 2012). Biological carbon fixation is the most direct and effective way to sequester CO_2_ in terrestrial ecosystems, and the main organisms that can fix CO_2_ are plants and autotrophic microorganisms (Liu et al., 2018). Among them, autotrophic microorganisms are widely distributed and have strong environmental adaptability in converting CO_2_ into soil organic carbon to regulate atmospheric CO_2_ concentration and increase soil carbon sequestration (Ge et al., 2013). Some studies have shown that the carbon assimilation by microorganisms in wetlands, grasslands, and forests ranges from 10.3 to 40.1 mg kg^–1^ in 15 days (Lynn et al., 2016), and from the perspective of the material cycle and energy flow of the entire biosphere, CO_2_ fixation by microorganisms is a biological carbon fixation force that cannot be ignored (Elsaied and Naganuma, 2001; Nanba et al., 2004). Therefore, it is of great scientific importance to study the ecological and environmental effects of CO_2_ fixation by autotrophic microorganisms. The Calvin cycle is one of the key pathways for CO_2_ fixation by autotrophic soil bacteria, with the highest carbon sequestration efficiency. This enzyme-catalyzed cycle is catalyzed by Ribulose-1,5-bisphosphate carboxylase or oxygenase (RubisCO), which is encoded by the *cbbL* gene (Tabita, 1999). Therefore, the *cbbL* gene has been widely used by many scholars as a biomarker for the ecological characterization of autotrophic bacteria in soil environments of different ecosystems, including agricultural fields and lakes (Elsaied and Naganuma, 2001; Ge et al., 2013).

With an average altitude over 4,000 m, the Qinghai–Tibet Plateau (QTP) is known as the “roof of the world” and the “third pole.” Due to its unique geographical location and climatic conditions, it has formed a typical alpine grassland ecosystem. The alpine meadows cover an area of 1.28 × 10^6^ km^2^, which is the largest alpine meadows in China (Zhang et al., 2002). The unique biogeochemical processes and fragile ecological environment of alpine meadows make them more sensitive to global climate change and anthropogenic disturbances (Lan, 2004; Wischnewski et al., 2011) and have been ideal sites for ecological studies of diversity distribution patterns. In recent years, the alpine meadow ecosystem has been severely damaged by natural and anthropogenic factors, with large areas of good grassland degraded to bare ground or “heitutan,” and the phenomenon of grassland desertification and salinization intensified (Wang et al., 2005), especially in the Sanjiangyuan area in the center of QTP (Chong and Zhang, 2015; Shao et al., 2017). Heitutan is characterized by increased bare land proportion, reduced edible herbage, and commensurate increases in the dominance of unpalatable species (Li et al., 2014). Nearly 4,908,900 hectare of alpine meadows have been degraded to heitutan, with 3,122,400 hectare of degraded heitutan (slopes < 7°) on flat areas, 1,533,800 hectare on gentle slopes (7° ≤ slopes < 25°), and 252,800 hectare on steep slopes (25° ≤ slopes) (Dong et al., 2015). In contrast, alpine meadow soils store large amounts of root and organic carbon, which is an important global carbon reservoir and profoundly affects the global terrestrial ecosystem carbon cycle (Fang et al., 1996; Wang L. et al., 2019). The degradation of alpine meadows will undoubtedly have a negative impact on the ecological security of the QTP and even the world (Wang et al., 2021). Therefore, effective measures must be taken to control degraded alpine meadows.

Nitrogen (N) is the most important nutrient limiting plant growth in many ecosystems, and N addition is the most common management to promote productivity in degraded grasslands and maintain nutrient balance in grassland ecosystems (Wang et al., 2017). Exogenous N inputs can increase the amount of available N in the soil, alleviate or eliminate N limitation, change the chemical elemental composition of the soil, and promote plant growth (Verburg et al., 2010; Zhang et al., 2014; Chen et al., 2017). Soil microorganisms have rapid reproduction rates and respond rapidly to soil physicochemical properties changes (Wang et al., 2022), and N addition can affect the composition, structure, and function of soil microbial communities (Wang et al., 2014; Liu H. M. et al., 2017). For example, Yuan et al. (2012) found that long-term fertilization had significant effects on the structure, diversity, and abundance of soil carbon-fixing bacteria revealing the effects of different fertilization regimes (NPK and NPK with straw return) on soil carbon-fixing bacteria. Wang R. et al. (2019) studied changes in the abundance and structure of carbon-fixing bacterial communities in white pulp soils after the conversion of forest land to cropland in the hilly areas of Northeast China and showed that fertilizer application (NPK compound fertilizer) caused changes in the abundance and diversity of carbon-fixing bacterial communities. Zhou et al. (2019) studied the effects of long-term chemical fertilizer regimes (NP, NK, PK, and NPK) on the activity and community composition of soil autotrophic bacteria and showed that the application of chemical fertilizers altered the ecological characteristics of carbon-fixing bacteria and had a significant effect on the carbon-fixing bacteria diversity. Selesi et al. (2005) found significant changes in carbon-fixing bacterial communities’ composition and diversity after the application of mineral fertilizers and mixed compost in wheat field soils. Current studies have focused on changes in soil carbon-fixing bacterial communities after fertilizer application in soils such as paddy fields and arable land, and experimental results have also shown that the structure and diversity of carbon-fixing bacterial communities are sensitive to fertilizers. But the report of N addition effecting on carbon-fixing microorganisms in degraded alpine meadows is rare. In addition, due to the complex terrain characterized by the vertical and horizontal ravines in the Sanjiangyuan region, and the large distribution span of different types of terrain such as slope, the composition and diversity of carbon-fixing microbial community may vary in different slopes of the nitrogenous grassland. Rare studies compare carbon-fixing microbial community on different slopes. Therefore, it is of great scientific significance to study the effects of N addition on soil carbon-fixing microorganisms on different slopes in degraded meadow ecosystems.

Thus, this study aims to answer the following two scientific questions to understand the alpine meadow soil carbon-fixing bacterial communities change rule and provide reference for scientific fertilization system of the degraded alpine meadow in the Sanjiangyuan region: (1) Is there any difference in the characteristics of carbon-fixing microorganisms between N addition levels on different hill slopes in the degraded alpine meadow? (2) What is the relationship between carbon-fixing microorganisms and slope, N addition, microbial biomass, and physicochemical properties?

## Materials and Methods

### Study Area

**FIGURE 1:**
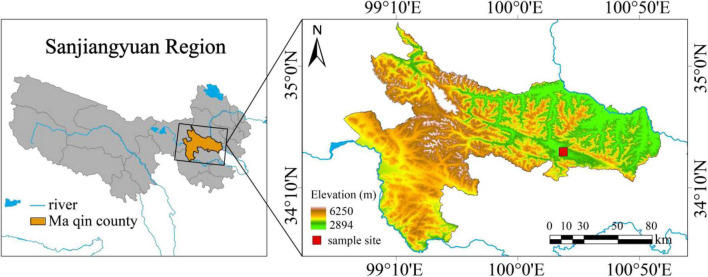
Location of the study site.

The sampling site is located in Dawu Town, Maqin County, Guoluo Tibetan Autonomous Prefecture, Qinghai Province, west China (34°25′20.41″ N, 100°19′55.72″ E, average altitude 3,768 m) (Figure 1), with a continental cold climate. The average annual temperature is −3.9 to −3.5°C, and the average annual precipitation is 423–565 mm. The surface soil is about 4–10 cm of turf layer, and it belongs to a type of alpine meadow soil. Due to the disturbance of overgrazing, rodent activity, and climate change, this place has become a moderate degraded alpine grassland with vegetation coverage less than 80%. The uncontrolled grazing all year round. The plant community composition of the test sites on the gentle slope and steep slope is basically the same, consisting of *Kobresia humilis*, *Kobresia pygmaea*, *Poa pratensis*, *Elymus nutans*, *Ajania tenuifolia*, *Ligularia virgaurea*, *Aconitum flavum*, *Oplopanax elatus*, *Euphorbia fischeriana*, etceteras.

### Experimental Design

**FIGURE 2:**
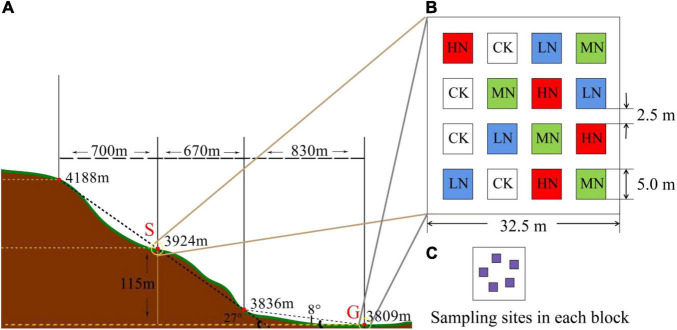
**(A)** The distribution of gentle slope area and steep slope area. **(B)** The distribution of fertilization plots. **(C)** Sampling sites in each block.

In early May 2019, a square of 50 m × 50 m was selected as the sampling sites in steep slope (S) and gentle slope (G) with the slope angle of 27° and 8°, respectively (Figure 2A). The aspect of the sampling slope was 12.3° from west to north. A randomized block experimental design was adopted, and 16 quadrats (5 m × 5 m) were set up in two sampling areas, a total of 32 quadrats were set up to conduct N addition experiments with four levels including low equal nitrogen supplemental level (LN): 2 g N⋅m^–2^⋅a^–1^, middle nitrogen supplemental level (MN): 5 g N⋅m^–2^⋅a^–1^, high equal amount of nitrogen (HN): 10 g N⋅m^–2^⋅a^–1^, and 0 g N⋅m^–2^⋅a^–1^ served as the control (CK). Buffer zone between quadrats was 2.5 m (Figure 2B). After the plot was established, nitrogen supplement (NH_4_NO_3_, grain loading, nitrogen content 35%) was added when the weather was not rainy to reduce fertilizer loss.

### Sample Analysis

#### Soil Sample Collection

In May 2019, the first fertilization was applied. In May 2020, fertilization was applied again in the second year. In August 2020, samples were collected in five plots (10 cm × 10 cm) when herbage was growing vigorously (Figure 2C). Sampling tools were disinfected with alcohol, soil samples with the same weight were collected from 0 to 10 cm soil layer using a soil corer (0–10 cm deep with a 3 cm inner diameter), and then they were mixed to obtain one representative composite sample (totally 32 samples). After removing impurities such as roots, a certain amount of soil was put into an aseptic tube. Approximately 10 g of each sample was collected and quickly frozen in a tank of liquid N. After being brought back to the laboratory, it was immediately sealed and transported for sample testing with a large volume of dry ice. We entrusted the determination of soil samples *cbbL* gene to Beijing Allwegene Technology Co., Ltd. The remaining soil samples were brought back to the laboratory. The natural air drying and grinding and screening were conducted for soil physicochemical properties and microbial biomass analysis (Supplementary Tables 1, 2 for the determination methods and values).

#### Soil Microbial Community Structure and Diversity Analysis

The gene primers were *cbbL*-F: 5′-GACTTCACCAAAGA CGACGA-3′ and *cbbL*-R: 5′-TCGAACTTGATTTCTTTCCAC-3′; *cbbL*-F: 5′-CATCATGTTCGACCAGGACT-3′ and *cbbL*-R: 5′-TCGAACTTGATTTCTTTCCA-3′ (Ji et al., 2016). The test steps are: genomic DNA extraction—genomic DNA quality inspection—PCR amplification—PCR product electrophoresis detection—PCR product purification—Miseq library construction—Miseq library quality inspection—Illumina Miseq machine sequencing platform.

##### DNA Extraction

Soil microbiome DNA was extracted using the PowerSoil DNA Isolation Kit (MoBio Laboratories, Inc., CA) [Omega E.Z.N.A. Stool DNA Kit (Omega Bio-tek, Inc., Beijing, China)] kit instructions. The extracted DNA was assayed for DNA quality and concentration using a Nanodrop 2000 (Thermo Fisher Scientific, Inc., MA, United States). The samples were stored at -20°C for subsequent experiments.

##### PCR Amplification

PCR reaction system (total system is 25 μL): 12.5 μL 2xTaq Plus Master Mix II (Vazyme Biotech Co., Ltd., China), 3 μL BSA (2 ng/μL), 1 μL Forward Primer (5 μM), 1 μL Reverse Reaction parameters: 95°C pre-denaturation for 5 min; denaturation at 95°C for 45 s, annealing at 55°C for 50 s, extension at 72°C for 45 s, 28 cycles; extension at 72°C for 10 min. The PCR products were amplified using 1% agarose gel electrophoresis to detect the size of the amplified target bands and purified using the Agencourt AMPure XP (Beckman Coulter, Inc., FL, United States) nucleic acid purification kit.

##### MiSeq Sequencing

PCR products were used to construct microbial diversity sequencing libraries using the NEB Next Ultra II DNA Library Prep Kit (New England Biolabs, Inc., MA, United States) library builder kit. Paired-end sequencing was performed at Beijing Allwegene Technology Co., Ltd., Beijing, China using the Illumina Miseq PE300 (Illumina, Inc., CA, United States) high-throughput sequencing platform. The sequenced raw sequences were uploaded to NCBI’s SRA database.

In order to make the analysis results more accurate and reliable, after the sequencing raw data were launched, data quality control was first performed to obtain optimized sequences through sequence splicing, filtering, and chimera removal, and then OTUs (operational taxonomic units) clustering and annotation were performed. Bioinformatic statistical analysis of OTUs at 97% similarity level was performed using the UPARSE method (Edgar, 2013). Based on the clustering results, diversity analysis could be performed; the OTUs were annotated using the Silva (Release 128/132)^1^ database (Quast et al., 2012), and based on the annotation results, species information for each taxon could be obtained, allowing correlation analysis of sample composition and differences in community outcomes between samples.

Alpha diversity analysis includes Chao1, Observed_species, and Shannon, to estimate the species abundance and diversity of environmental communities.

Beta diversity analysis (non-metric multidimensional scaling method, NMDS) focuses on differences in microbial community composition between samples (Rivas et al., 2013).

### Data Statistics

The analysis platform of this study is Qiime platform^2^, and Kruskal–Wallis test is carried out in R programming (qvalue package) to analyze the N addition treatments for the same slope as well as OTUs of soil carbon-fixing microorganisms and the abundance differences of carbon-fixing bacteria phyla and genus in different slopes.

One-way ANOVA and Duncan are conducted by SPSS Statistics 20.0 statistical analysis software to analyze the effects of different N addition treatments on soil physicochemical properties, stoichiometric proportion, microbial biomass, Chao1 index, observed species number, and Shannon index for the same slope. Independent sample *t*-test is used to analyze the differences of soil physicochemical properties, stoichiometric proportion, microbial biomass, Chao1 index, observed species number, and Shannon index between different slopes (α = 0.05).

Venn diagram and species composition histogram was calculated and plotted by R language tool. The dilution curve was analyzed by mothur based on OTUs clustering results. For NMDS analysis, vegan and ggplot2 packages in R were used for data calculation and mapping. Network analysis was accomplished by R language package and psych package. Mantel test and Pearson correlation analysis were performed with R language ggplot2, ggcor, and dplyr software packages.

## Results

### Abundance of Soil Carbon-Fixing Bacteria Species

**FIGURE 3:**
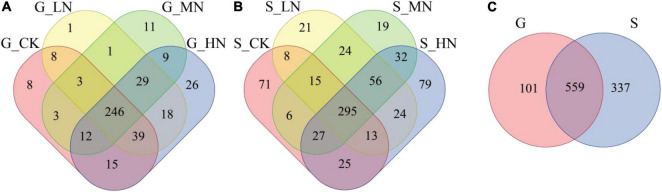
Venn diagrams of different slopes and different nitrogen addition treatments (**A**, the number OTU on gentle slope; **B**, the number of OTU on steep slope; **C**, total OTU number for both gentle slope and steep slope).

On gentle slope, the number of OTUs common to all treatment soil samples was 246, accounting for 57.3%, while the number of OTUs specific to CK, LN, MN, and HN was 8, 1, 11, and 26, accounting for 1.9, 0.2, 2.6, and 6.1%, respectively (Figure 3A). On steep slope, the number of OTUs common to all treatment soil samples was 295, accounting for 41.3%, while the number of OTUs specific to CK, LN, MN, and HN was 71, 21, 19, and 79, accounting for 9.9, 2.9, 2.7, and 11.0%, respectively (Figure 3B). The number of OTUs shared between the gentle slope and steep slope soil samples was 559, a proportion of 56.1%, with 101 OTUs specific to the gentle slope and 337 OTUs specific to the steep slope (Figure 3C). The Kruskal-Wallis test for OTUs showed that 27 OTUs differed significantly (*P* < 0.05) between N application treatments on gentle slope, 22 OTUs differed (*P* < 0.05) between N application treatments on steep slope, and 450 OTUs differed significantly (*P* < 0.05) between gentle slope and steep slope (Supplementary Table 3). A total of 7 Phylum, 11 Classes, 22 Orders, 42 Families, 76 Genus, and 109 Species of carbon-fixing microbial OTUs were identified in the taxonomic lineage.

### Community Composition and Relative Abundance of Soil Carbon-Fixing Bacteria

Most of these microorganisms could only be classified in the bacterial groups of the higher taxonomic levels, Proteobacteria and Actinobacteria. Using the phylum as the taxonomic level (Supplementary Figure 1), Proteobacteria was the dominant phylum, with the relative abundance of Proteobacteria above 93.0% in all but one sample, SCK_4. At the genus level (Supplementary Figure 2), *Sulfurifustis* was the dominant genus with relative abundances ranging from 16.5 to 90.9%, respectively, with an average abundance of 56.5%. The Kruskal–Wallis test showed that at the phylum level, the variation in abundance of each phylum of N-added carbon-fixing bacteria on the same slope was not significant. Only Cyanobacteria with mean abundance <1% differed between gentle slope and steep slope (*P* < 0.05) (Supplementary Table 4). At the genus level, the dominant genera were not significantly different between treatments on the same slope and between slopes, but some low abundance genera of carbon-fixing bacteria (<0.5% relative abundance) were significantly different (*P* < 0.05). For example, N addition differed in *Thiorhodococcus*, *Rhodovulum*, *Serpentinomonas* on gentle slope and in *Ectothiorhodospira*, *Sulfuricaulis*, *Cupriavidus* on steep slope (*P* < 0.05). The results of the Kruskal–Wallis one-way AVOVA multiple comparison test for differences between treatments are shown in Supplementary Tables 5, 6. On gentle slope, HN significantly increased the relative abundance of *Rhodovulum* compared to the control (*P* < 0.05). On steep slope, the relative abundance of Ectothiorhodospira was significantly increased (*P* < 0.05) under MN and HN and *Cupriavidus* was significantly increased (*P* < 0.05) under HN compared to the control. There were significant differences (*P* < 0.05) between slopes in the 28 genera of identified carbon-fixing bacteria. The relative abundance of *Cupriavidus* and *Alkalispirillum* was significantly higher on steep slopes than on gentle slope, while the relative abundance of *Rhodovulum*. was significantly lower than on gentle slope (Supplementary Table 7). This indicates that the effects of N addition and slope on the dominant phylum and genus of carbon-fixing bacteria in degraded alpine meadows were not significant, but only on some low abundance phylum and genus of carbon-fixing bacteria.

### Microbial Network Correlation

**FIGURE 4:**
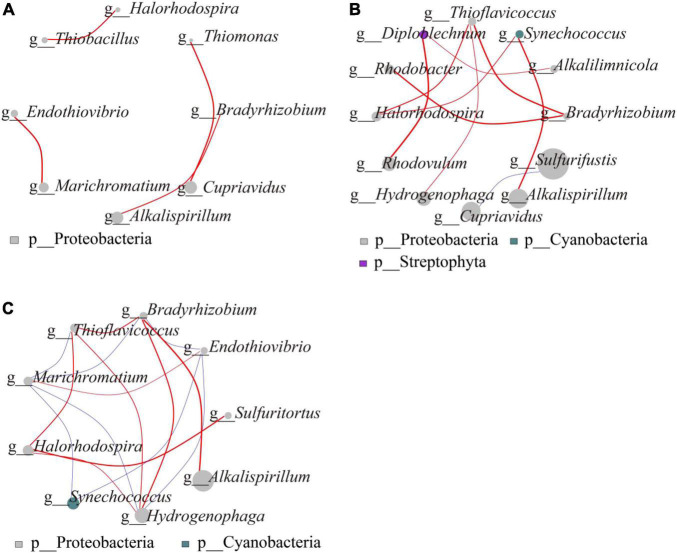
Microbial network interaction of carbon-fixing microorganisms (**A**, microbial network interaction on gentle slope; **B**, microbial network interaction on steep slopes; **C**, network interaction between gentle slope and steep slope). The size of the dot in the graph represents the abundance and the thickness of the line represents the correlation; the color of the dot represents the door to which it belongs, the red line represents a positive correlation, and the blue line represents a negative correlation.

The microbial network interactions map shows the complex relationships consisting of highly linked genera, which on gentle slope (Figure 4A) form four modules, each belonging to the Proteobacteria. The first module consisted of *Thiobacillus* and *Halorhodospira*, the second, third and fourth modules consisted of *Thiomonas* and *Cupriavidus*, *Bradyrhizobium* and *Alkalispirillum*, *Marichromatium*, and *Endothiovibrio*, respectively, with positive correlations between them (*P* < 0.05). On steep slope (Figure 4B), *Diploblechnum* belonged to Streptophyta, *Synechococcus* to Cyanobacteria and the other 10 genera to Proteobacteria. Of these, *Cupriavidus*, which differed significantly between the different N addition treatments, was negatively correlated with the dominant genus *Sulfurifustis* (*P* < 0.05). All other genera were positively correlated with each other. The network interactions between gentle slope and steep slope showed that *Alkalispirillum* and *Bradyrhizobium*, which differed significantly, were positively correlated (*P* < 0.05) (Figure 4C).

### Carbon-Fixing Community Diversity

**FIGURE 5:**
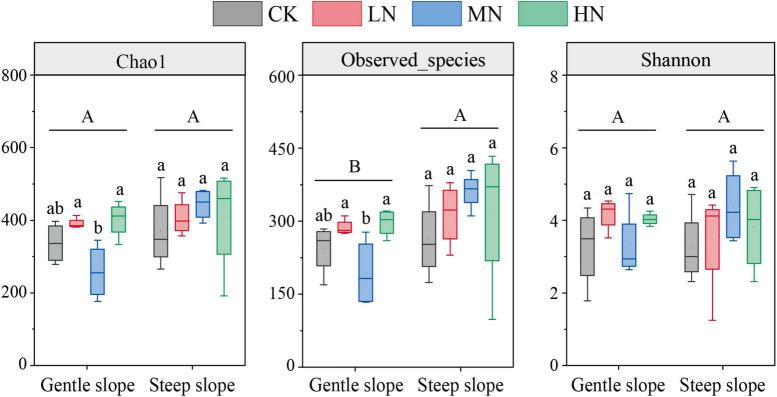
Abundance and diversity index of carbon-fixing microbial community in soil samples. Different lowercase letters represent significant differences among treatments in the same slope, and different uppercase letters represent significant differences among slopes.

**FIGURE 6:**
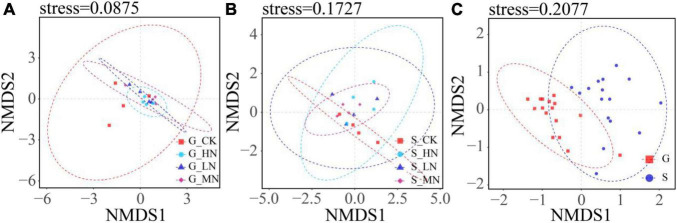
NMDS analysis of OTU level (**A**, groups of nitrogen addition on gentle slope; **B**, groups of nitrogen addition on steep slope; **C**, groups of gentle slope and steep slope). If the soils in the same group are in a circle, it means that the difference between groups is not obvious, and the non-intersection of circles between groups means that there is a certain difference between the groups.

The results of the one-way ANOVA showed that MN significantly reduced the Chao1 richness and observed species values of carbon-fixing bacteria on gentle slope (*P* < 0.05). Whereas N addition had no significant effect on Chao1 index, observed species number and Shannon diversity on steep slope (Figure 5). Independent sample *t*-tests showed that the observed species number was significantly higher on steep slope than on gentle slope (*P* < 0.05). The results of NMDS showed that different treatments on the same slope and different treatment groups on different slopes intersected with each other, indicating that none of the differences in soil carbon-fixing microorganisms between groups were significant (Figure 6).

### Comparison of Gentle Slope/Steep Slope Soil in CK

Under natural conditions (CK), There were significant differences in TK, MBC and soil C:N between gentle slope and steep slope. Other soil nutrients, stoichiometric ratio, community composition, and diversity were similar (Supplementary Table 8).

### Carbon-Fixing Bacterial Communities in Relation to Microbial Biomass and Soil Physicochemical Properties

**FIGURE 7:**
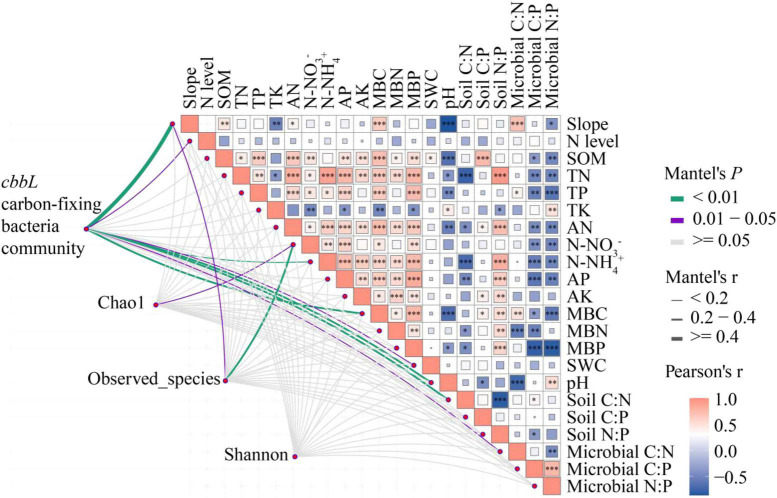
Correlation of carbon-fixing microbial community and diversity with slope, nitrogen addition level, soil physicochemical properties, stoichiometric proportion, and microbial biomass. Mantel edge width corresponds to Mantel r value, and edge color indicates statistical significance. The color gradient of Pearson correlation coefficient r represents the paired correlation of variables. 27° for steep slope and 8° for gentle slope. N addition levels include CK: 0 g N⋅m^–2^, LN: 2 g N⋅m^–2^, MN: 5 g N⋅m^–2^, and HN: 10 g N⋅m^–2^. Carbon-fixing microbial community includes 28 bacteria genera with significant differences among different slopes. * indicates 0.01 < *P* < 0.05, ^**^ indicates *P* < 0.01, and ^***^ indicates *P* < 0.001.

Pearson correlation analysis showed significant positive correlations between slope and SOM (*r* = 0.454, *P* = 0.009), AN (*r* = 0.369, *P* = 0.038), MBC (*r* = 0.630, *P* = 0.000), and microbial C:N (*r* = 0.701, *P* = 0.000), and significant negative correlations with TK (*r* = −0.542, *P* = 0.001), pH (*r* = −0.867, *P* = 0.000), and microbial N:P (*r* = −0.417, *P* = 0.018) (Figure 7). There was no significant correlation between the level of N addition and the physicochemical properties and microbial biomass of the soil. The results of Mantel test analysis showed that slope, N level, N-NH_4_^+^, MBC, SWC, pH, soil C:N, microbial C:N, and carbon-fixing bacteria community abundance were significantly positively correlated (*P* < 0.05). N-NO_3_^–^ and carbon-fixing bacteria Chao1 index were significantly positively correlated (*P* < 0.05). Slope, N-NO_3_^–^ and the observed number were significantly positively correlated (*P* < 0.05). There was no significant correlation between slope, N level, soil physicochemical properties, microbial biomass, stoichiometric ratio, and Shannon diversity. This indicates that N addition at different slopes had a significant effect on the community structure and a non-significant effect on the diversity of carbon-fixing bacteria communities.

## Discussion

### Effects of Nitrogen Addition on the Community of Carbon-Fixing Bacteria

Carbon sequestration by autotrophic microorganisms is the key to biogeochemical carbon cycling in soil ecosystems. In this paper, we compare the differences in the structure and diversity of soil carbon-fixing bacterial communities at different slopes after N addition in a degraded alpine meadow. We found that among the carbon-fixing bacterial communities, at the phylum level, Proteobacteria was the most abundant phylum belonging to the studied groups after N addition on both gentle and steep slopes and accounted for the largest proportion of the soil, which is consistent with the findings of previous studies (Gao et al., 2018). The strong environmental adaptability, rapid growth and reproduction, and the ability to take up substrate are the main reasons for the dominance of Proteobacteria in degraded alpine meadows (Salcher et al., 2010; Simek et al., 2011). The variation in abundance of each phylum of N-added carbon-fixing bacteria on the same slope was not significant. At the genus level, *Sulfurifustis* was the dominant genus. The dominant genus was not significantly different between the different N additions on the same slope, but N addition had a significant effect on some low abundance carbon-fixing genera, and the response of carbon-fixing microorganisms in soils varied between slopes after N application. This indicates that the degree of species specificity of carbon-fixing microorganisms in degraded alpine meadow soils is low under N addition. In terms of the effect of N addition on the abundance and diversity of carbon-fixing bacteria, middle N addition in gentle slope significantly reduced the Chao1 and observed species values, while any horizontal N addition on steep slope had no significant effect on Chao1 index, observed species numbers, and Shannon diversity. Our previous results comparing the relationship between N addition and soil bacterial community diversity showed that soil bacterial richness and diversity tended to decrease and then increase with increasing N application, with moderate N addition significantly reducing soil bacterial richness and diversity, and high levels of N addition significantly inhibiting the decrease in soil bacterial richness and diversity (Li et al., 2020a). The results of the Mantel test showed a significant positive correlation between N level and carbon-fixing bacteria community abundance, and no effect of N level on Shannon diversity. The results of the Mantel test showed that N level and carbon-fixing bacteria community abundance were significantly and positively correlated. High levels of 10 g⋅m^–2^ N addition suppressed significant decreases in soil carbon-fixing bacteria abundance and observed species numbers, and could maintain soil ecosystem stability.

### Effects of Slope on the Community of Carbon-Fixing Bacteria

There were no significant differences in soil microorganisms community structure and diversity in CK between gentle slope and steep slope, but there were significant differences in soil total potassium and microbiomass carbon. Compared to CK, the abundance of Cyanobacteria, organic matter, alkaline nitrogen, pH, soil C:N ratio, microbial C:N ratio, microbial N:P ratio, and the number of carbon-fixing bacteria species changed between the gentle slope and steep slope after N addition. The soil C:N ratio changed from significant to non-significant, and the abundance of Cyanobacteria, organic matter, alkaline nitrogen, microbial biomass C:N ratio, and number of species were significantly higher on steep slope than that on gentle slope, while pH and microbial biomass N:P ratio were significantly higher on gentle slope than that on steep slope. The variability between slopes after N application may be since slope is interfered with by microhabitat and microclimatic conditions such as light, which has an important influence on soil thickness, soil moisture content, and the degree of soil erosion in grassland (Zhang, 2019), resulting in soil total potassium and microbial carbon showing heterogeneity across slopes. The results of the Mantel test showed that slope and carbon-fixing bacteria community abundance and number of species observed were significantly positively correlated, with no significant effect on diversity. This indicates that the differences in carbon-fixing bacteria communities are related to slope, while Shannon diversity is not related to slope. In combination with the results of this study the three N addition levels on steep slopes had no significant effect on soil physicochemical properties, microbial biomass, stoichiometric ratios, or on the number of carbon-fixing bacteria Chao1, observed species, or diversity. The promoting effect of exogenous N addition was slope dependent and more appropriate for vegetation and soil restoration in degraded alpine grasslands on gentle slope, both for vegetation restoration (Li et al., 2020b) and for soil carbon-fixing bacteria richness and species number. There is an interdependent and competitive relationship between soil nutrients and plants. Nutrients play an important role in the growth and development of plants and the succession of community structures and will directly affect plant nutrient uptake and plant growth, which may ultimately lead to differences in plant community productivity. Plant growth is accelerated when little N is applied, and most of the N applied to the soil may be taken up by plants, resulting in microorganisms not receiving enough N for their own reproduction, and therefore medium N leads to a decrease in microbial abundance and species numbers, thus affecting the stability of grassland systems. When N application is increased, the effective amount of soil nutrients is altered, the competition for nutrients between microbes and plants is weakened and carbon-fixing microbes may consume more of the fast-acting nutrients for their own reproduction. This leads to an increase in the abundance and number of species of carbon-fixing microorganisms under high N conditions. The need to mix other fertilizers with N application to maintain soil nutrient balance needs to be verified by further research.

### Limiting Factors for Carbon-Fixing Microorganisms

There is a correlation between soil microbial community structure and soil physicochemical properties (Liu Q. et al., 2017). For example, it has been suggested that there is a correlation between soil carbon-fixing bacterial communities and all physicochemical properties of the soil, with soil pH and total N having the most significant effect on bacterial communities (Su et al., 2020). Li et al. (2020c) showed that soil pH, SOC, and bulk weight could help explain the changes in soil microbial composition observed in the study and that changes in soil fast-acting nutrients such as N-NO_3_^–^, AK, and AP had a significant effect on the abundance and diversity of carbon-fixing bacteria. The results of the Mantel test in this study showed a significant positive correlation between N-NH_4_^+^, MBC, SWC, pH, soil C:N, microbial C:N, and carbon-fixing bacterial community abundance, a significant positive correlation between N-NO_3_^–^ and Chao1 index, and a significant positive correlation between N-NO_3_^–^ and observed species number. Soil physicochemical properties, microbial biomass, and Shannon diversity of carbon-fixing bacteria were not significantly correlated, indicating that the contribution of the fast-acting nutrient N-NO_3_^–^ to CO_2_ fixing microorganisms was significant. In addition to the influence of soil physicochemical properties on microbial community structure and diversity, an increasing number of studies have shown that soil C, N, and P ratios are the main drivers of microbial diversity (Zhang et al., 2016; Yang et al., 2020, 2022). The present study also showed that carbon-fixing bacterial community abundance and soil and microbial C:N:P ratios were closely related. Specifically, soil and microbial C:N were strongly related to the carbon-fixing bacterial community, while soil and microbial C:P and N:P were weakly related to the carbon-fixing bacterial community. This suggests that soil and microbial C:N ratios have a greater effect on carbon-fixing bacteria. However, stoichiometric ratios had no significant effect on the Shannon diversity of carbon-fixing bacteria.

Our short-term results can reflect the transient kinetic response of alpine meadows to N addition. Whether these short-term results can be translated to longer time scales needs to be tested. In future studies, in addition to the Calvin cycle, some other CO_2_ fixation pathways of autotrophic microorganisms should be considered, such as the reduced tricarboxylic acid cycle, the reductive acetyl CoA pathway, 3-hydroxypropionic acid cycle, 3-hydroxypropionic acid/4-hydroxybutyric acid cycle, and the 2-carboxylic acid/4-hydroxybutyric acid cycle (Thauer, 2007; Berg, 2011; Jeoung et al., 2014). Quantitative studies of key genes and their expression profiles in these biological carbon sequestration processes are needed to understand the specific contribution of these microorganisms to CO_2_ fixation.

## Conclusion

The dominant species composition of the soil carbon-fixing bacterial community in degraded alpine meadows on gentle/steep slopes after N addition was similar, with Amoeba being the most abundant phylum in the study and *Sulfurifustis* being the dominant genus. Exogenous N addition had a significant effect on soil nutrients (TK, AP), microbial biomass (P), stoichiometric ratios (soil C:N, microbial C:N, microbial C:P) and the abundance and number of species of carbon-fixing bacteria in degraded alpine meadows, and this effect was dependent on slope positions. Significant differences in abundance of Cyanobacteria and 28 genera of identified carbon-fixing bacteria, soil nutrients (SOM, TK, AN), microbial biomass (C), pH, and stoichiometric ratios (microbial C:N, microbial N:P) were found between gentle and steep slope after N addition. This result highlights the need for future research to integrate the effects of topographic factors on different degraded grasslands to better understand the mechanisms of degraded grassland restoration and management. For the management of degraded alpine meadows on steep slopes, N addition is not recommended for restoration, and the corresponding restoration and management options need to be studied in depth.

## Data Availability Statement

The raw sequence data from this study were deposited in the NCBI database with the study accession number: PRJNA843979 that are publicly accessible at http://www.ncbi.nlm.nih.gov/bioproject/843979.

## Author Contributions

CL carried out the field experiments and sampling and analyzed the data. CL wrote the original manuscript, with contributions from XL, YS, HL, and YY. All authors gave approval to the final version of the manuscript.

## Conflict of Interest

The authors declare that the research was conducted in the absence of any commercial or financial relationships that could be construed as a potential conflict of interest.

## Publisher’s Note

All claims expressed in this article are solely those of the authors and do not necessarily represent those of their affiliated organizations, or those of the publisher, the editors and the reviewers. Any product that may be evaluated in this article, or claim that may be made by its manufacturer, is not guaranteed or endorsed by the publisher.

## Footnotes

1
https://www.arb-silva.de/

2
http://qiime.org/scripts/assign_taxonomy.html
