# Supplementary material for: Effects of Nitrogen Addition on Soil Carbon-Fixing Microbial Diversity on Different Slopes in a Degraded Alpine Meadow
Source: Front Plant Sci. 2022 Jun 24;13:921278. doi: 10.3389/fpls.2022.921278 (PMC9263980; doi:10.3389/fpls.2022.921278)
Supplement: Supplementary file 1 [file Data_Sheet_1.docx]

**Effects of Nitrogen Addition on Soil Carbon-fixing Microbial Diversity on Different Slopes in a Degraded Alpine Meadow**

Chengyi Li^1^, Xilai Li^1, 2,^ *, Yan Shi^3^, Yuanwu Yang^1, 2^, Honglin Li^2^

^1^ College of Agriculture and Animal Husbandry, Qinghai University, Xining 810016, China.

^2^ State Key Laboratory of Plateau Ecology and Agriculture, Qinghai University, Xining 810016, China.

^3^ School of Environment, the University of Auckland, Auckland 1010, New Zealand.

* Corresponding author

E-mail address: [xilai-li@163.com](mailto:xilai-li@163.com) (X.L.Li)

**SUPPLEMENTARY MATERIAL**

**1.1 Supplementary Figures**


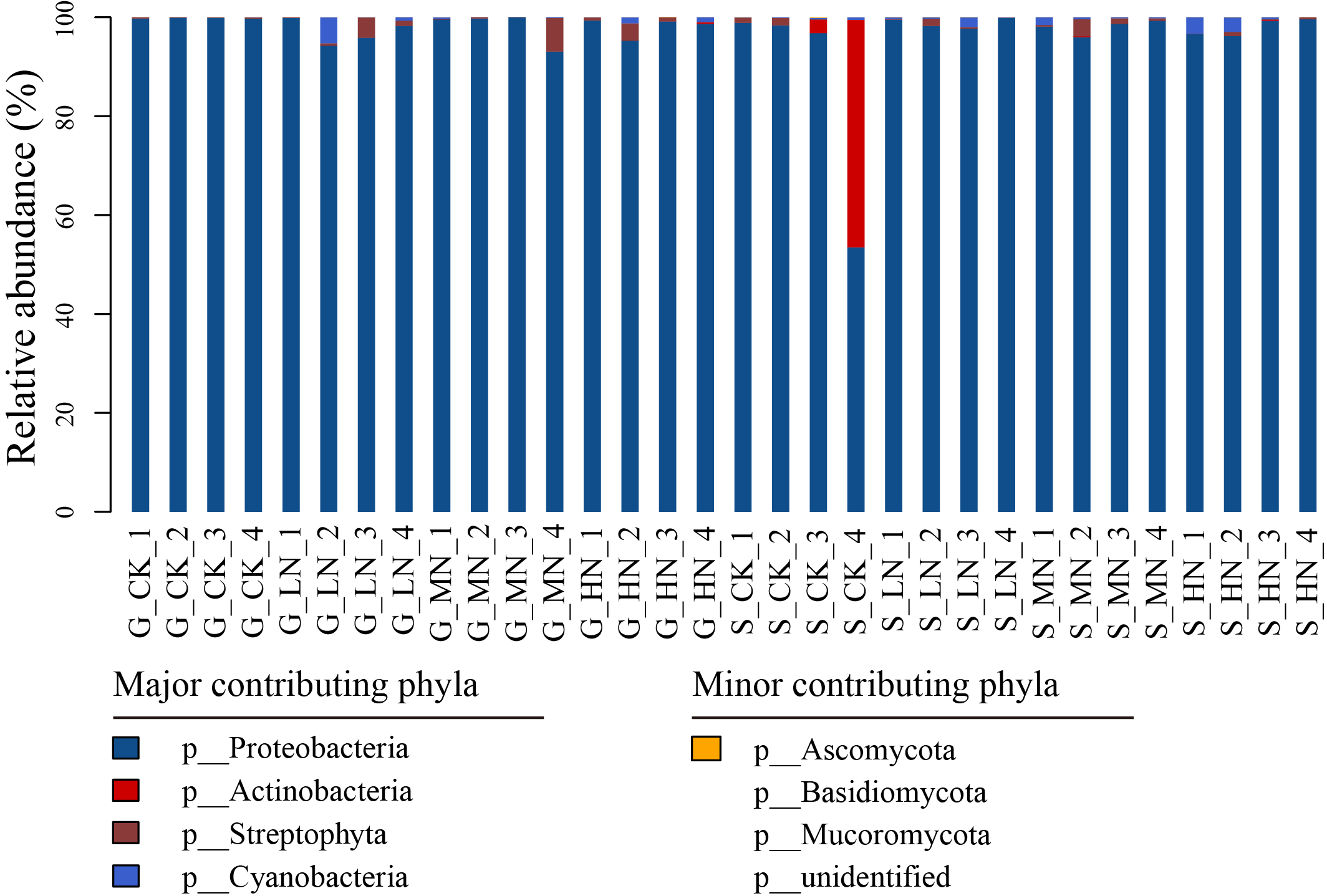


**SUPPLEMENTARY FIGURE 1** Species composition of soil carbon-fixing bacteria on different slopes with different nitrogen addition levels


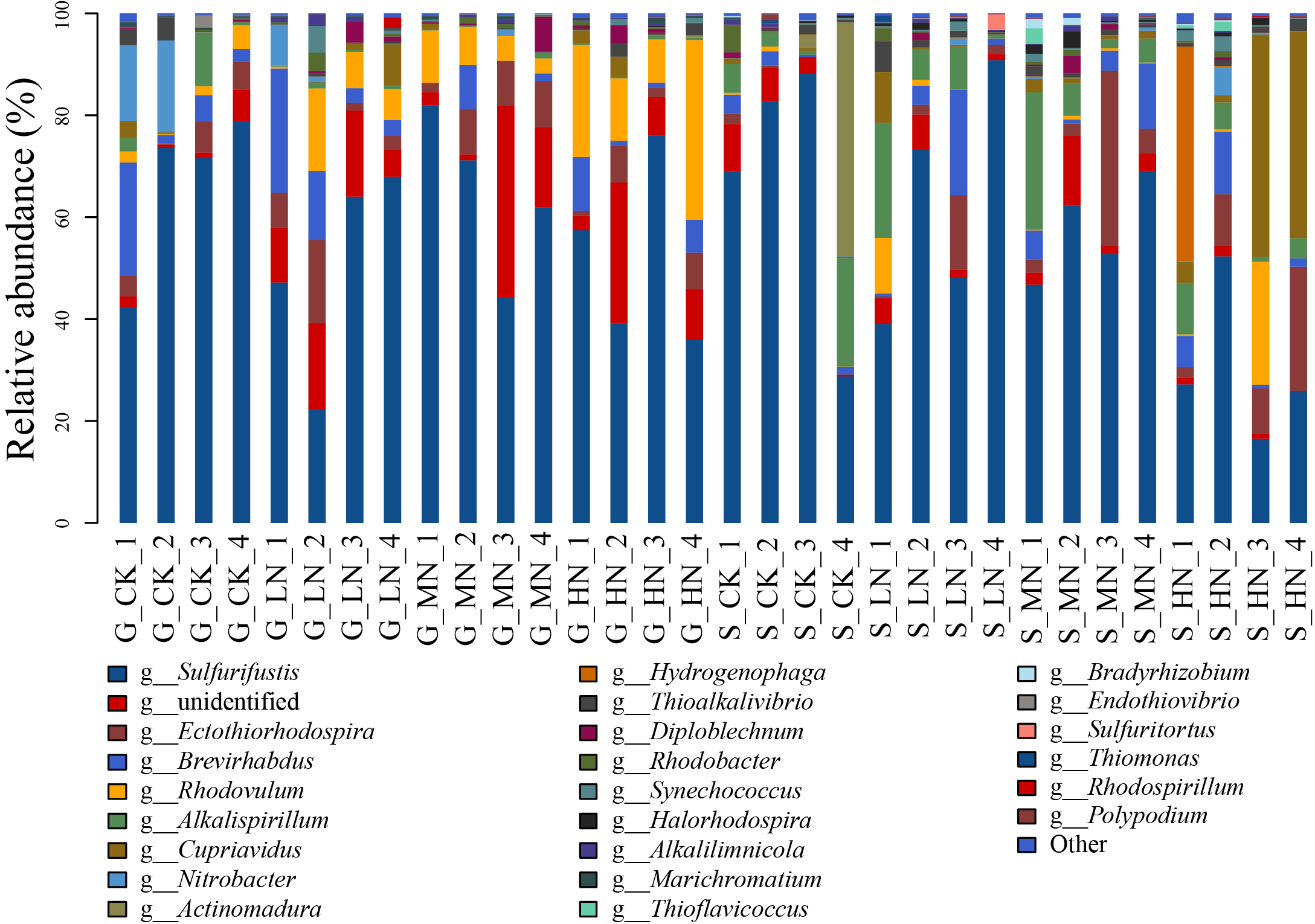


**SUPPLEMENTARY FIGURE 2** Species composition of soil carbon-fixing bacteria with nitrogen addition in different slopes at horizontal level

**1.2 Supplementary Tables**

**SUPPLEMENTARY TABLE 1** Soil Physical and Chemical Properties and Microbial Biomass measurment

| Parameters | Protocal |
| --- | --- |
| Soil organic matter (SOM) | Potassium dichromate volumetric method (external heating method) |
| Total nitrogen (TN) | Kjeldahl |
| Total phosphorus (TP) | Sodium hydroxide melting -- molybdenum-antimony resistance colorimetric |
| Total potassium (TK) | Sodium hydroxide melting -- flame photometry |
| Alkaline hydrolysis nitrogen (AN) | Alkaline hydrolysis diffusion |
| Nitrate nitrogen (N-NO_3_^-^) | Ultraviolet spectrophotometry |
| Ammonia nitrogen (N-NH_4_^+^) | Nannenberg reagent colorimetric |
| Available phosphorus (AP) | Sodium bicarbonate-Molybdenum-antimony resistance colorimetric |
| Available potassium (AK) | Acetic acid-flame spectrophotometry |
| Microbial biomass | Chloroform fumigation leaching |
| Soil water content (SWC) | Drying |
| pH | pHS-3C pH meter (soil: water = 1:2.5) |

**SUPPLEMENTARY TABLE 2** Soil microbial biomass, physicochemical properties, stoichiometric proportion under different slopes and different nitrogen additions

| Variable | Gentle slope | | | | | Steep slope | | | | |
| --- | --- | --- | --- | --- | --- | --- | --- | --- | --- | --- |
|  | CK | LN | MN | HN | Average | CK | LN | MN | HN | Average |
| SOM  (g/kg) | 48.30±9.52a | 43.01±2.02a | 47.04±3.61a | 40.73±6.15a | 44.77±6.24B | 55.35±9.74a | 49.94±7.69a | 53.26±6.95a | 47.15±2.25a | 51.42±7.21A |
| TN  (g/kg) | 3.11±0.60a | 2.78±0.17a | 3.08±0.21a | 2.52±0.44a | 2.87±0.43A | 3.18±0.45a | 3.02±0.40a | 3.09±0.35a | 4.02±2.42a | 3.33±1.20A |
| TP  (g/kg) | 0.62±0.08a | 0.58±0.02a | 0.61±0.01a | 0.58±0.03a | 0.60±0.04A | 0.64±0.06a | 0.67±0.17a | 0.62±0.04a | 0.62±0.06a | 0.64±0.09A |
| TK  (g/kg) | 22.76±0.85a | 19.14±0.52c | 18.14±0.28d | 20.79±0.43b | 20.21±1.88A | 17.69±0.61a | 18.92±0.98a | 18.76±0.52a | 18.00±1.28a | 18.34±0.96B |
| AN  (mg/kg) | 94.60±20.50a | 88.34±3.25a | 89.69±11.92a | 73.95±21.63a | 86.64±16.47B | 102.55±15.95a | 88.84±15.60a | 101.03±4.19a | 107.63±30.10a | 100.01±18.30A |
| N-NO_3_^-^  (mg/kg) | 17.41±4.72a | 19.19±5.17a | 26.66±8.29a | 16.09±4.03a | 19.84±6.68A | 21.70±7.72a | 21.87±7.94a | 23.15±7.25a | 23.47±8.22a | 22.54±7.01A |
| N-NH_4_^+^  (mg/kg) | 0.87±0.12a | 0.75±0.11a | 0.77±0.13a | 0.73±0.06a | 0.78±0.11A | 0.80±0.29a | 0.70±0.17a | 0.69±0.29a | 1.07±0.71a | 0.81±0.41A |
| AP  (mg/kg) | 3.35±0.95b | 2.56±0.15b | 5.19±1.39a | 3.07±1.52b | 3.54±1.45A | 4.03±1.34a | 4.63±2.50a | 4.67±2.42a | 4.67±4.27a | 4.50±2.55A |
| AK  (mg/kg) | 133.95±24.95a | 127.75±29.25a | 140.10±47.58a | 144.13±55.14a | 136.48±37.39A | 154.23±70.18a | 95.28±14.14a | 150.30±68.30a | 121.85±77.90a | 130.41±61.46A |
| MBC  (mg/kg) | 447.17±70.54a | 445.16±30.25a | 426.21±8.13a | 437.43±63.10a | 438.99±45.39B | 607.94±73.46a | 543.71±120.00a | 540.48±71.65a | 544.59±138.18a | 559.18±98.25A |
| MBN  (mg/kg) | 39.49±8.99a | 38.52±5.28a | 44.28±5.83a | 33.19±6.61a | 38.87±7.34A | 42.72±4.58a | 32.33±1.21a | 37.83±6.29a | 33.84±13.38a | 36.68±8.09A |
| MBP  (mg/kg) | 23.22±4.37a | 27.11±5.38a | 27.50±2.57a | 16.11±3.32b | 23.49±5.95A | 29.31±8.32a | 36.04±28.60a | 32.67±23.22a | 35.18±33.55a | 33.30±22.75A |
| SWC  (%) | 25.28±3.72a | 24.86±2.07a | 27.95±1.99a | 23.68±2.81a | 25.44±2.93A | 24.49±7.18a | 24.36±5.33a | 23.05±2.32a | 21.21±3.11a | 23.28±4.57A |
| pH | 7.72±0.52a | 8.23±0.05a | 8.20±0.11a | 8.23±0.28a | 8.09±0.35A | 7.11±0.34a | 7.08±0.42a | 7.07±0.08a | 6.98±0.14a | 7.06±0.26B |
| Soil C:N | 15.54±0.13b | 15.49±0.35b | 15.28±0.34b | 16.19±0.43a | 15.63±0.46A | 17.31±0.75a | 16.49±0.61a | 17.24±0.55a | 14.08±5.23a | 16.28±2.75A |
| Soil C:P | 77.49±9.89a | 73.81±4.24a | 77.73±6.30a | 70.22±7.90a | 74.81±7.33A | 86.72±12.18a | 75.76±11.73a | 85.45±6.79a | 75.87±6.41a | 80.95±10.14A |
| Soil N:P | 4.99±0.63a | 4.77±0.35a | 5.08±0.34a | 4.35±0.60a | 4.80±0.53A | 5.00±0.54a | 4.59±0.61a | 4.95±0.28a | 6.27±3.17a | 5.20±1.61A |
| Microbial C:N | 11.49±1.62ab | 11.64±0.81ab | 9.76±1.38b | 13.39±1.82a | 11.57±1.86B | 14.29±1.83a | 16.75±3.15a | 14.39±1.24a | 16.63±1.87a | 15.52±2.27A |
| Microbial C:P | 19.43±2.25b | 16.90±3.42b | 15.62±1.72b | 27.59±3.93a | 19.88±5.49A | 21.53±3.86a | 19.17±6.96a | 20.47±7.48a | 21.84±9.17a | 20.75±6.47A |
| Microbial N:P | 1.73±0.41a | 1.47±0.38a | 1.61±0.12a | 2.07±0.31a | 1.72±0.37A | 1.53±0.35a | 1.22±0.55a | 1.42±0.50a | 1.28±0.47a | 1.36±0.44B |

**Note:** CK is the control, LN, MN and HN are the low, medium and high levels of nitrogen addition treatment respectively. SOM: soil organic matter; TN: total nitrogen; TP: total phosphorus; TK: total potassium; AN: alkaline hydrolysis nitrogen; N-NO_3_^-^: nitrate nitrogen; N-NH_4_^+^: ammonia nitrogen; AP: available phosphorus; AK: available potassium; MBC: microbial biomass carbon; MBN: microbial biomass nitrogen; MBP: microbial biomass phosphorus; SWC: soil water content; pH: pH. Soil C:N represents the ratio of organic matter to total nitrogen; Soil C:P represents the ratio of organic matter to total phosphorus; Soil N:P represents the ratio of total nitrogen to total phosphorus; Microbial C:N represents the ratio of microbial biomass carbon to microbial biomass nitrogen; Microbial C:P represents the ratio of microbial biomass carbon to microbial biomass phosphorus; Microbial N:P represents the ratio of microbial biomass nitrogen to microbial biomass phosphorus. Different lowercase letters represent significant differences between treatments in the same slope, and different uppercase letters represent significant differences between slopes.

**SUPPLEMENTARY TABLE 3** Kruskal-Wallis test of OTU species with significant differences under different slopes and different nitrogen addition treatments

|  | OTU species |
| --- | --- |
| Different nitrogen addition treatments on gentle slope | OTU_108, OTU_139, OTU_165, OTU_180, OTU_19, OTU_197, OTU_213, OTU_228, OTU_24, OTU_245, OTU_257, OTU_286, OTU_303, OTU_319, OTU_34, OTU_348, OTU_360, OTU_365, OTU_382, OTU_394, OTU_401, OTU_404, OTU_45, OTU_54, OTU_6, OTU_7, OTU_90. |
| Different nitrogen addition treatments on steep slope | OTU_101, OTU_104, OTU_119, OTU_23, OTU_245, OTU_277, OTU_311, OTU_315, OTU_321, OTU_364, OTU_378, OTU_391, OTU_399, OTU_444, OTU_45, OTU_592, OTU_616, OTU_62, OTU_640, OTU_645, OTU_652, OTU_719. |
| Different slopes | OTU_1, OTU_100, OTU_1004, OTU_1006, OTU_101, OTU_1012, OTU_1020, OTU_103, OTU_106, OTU_107, OTU_108, OTU_11, OTU_111, OTU_114, OTU_115, OTU_116, OTU_119, OTU_121, OTU_124, OTU_125, OTU_126, OTU_127, OTU_128, OTU_130, OTU_131, OTU_132, OTU_134, OTU_138, OTU_139, OTU_14, OTU_140, OTU_143, OTU_144, OTU_149, OTU_151, OTU_153, OTU_156, OTU_157, OTU_158, OTU_159, OTU_163, OTU_164, OTU_166, OTU_167, OTU_169, OTU_175, OTU_176, OTU_178, OTU_179, OTU_180, OTU_182, OTU_183, OTU_188, OTU_189, OTU_190, OTU_191, OTU_192, OTU_195, OTU_197, OTU_198, OTU_199, OTU_20, OTU_204, OTU_205, OTU_206, OTU_207, OTU_209, OTU_21, OTU_210, OTU_215, OTU_222, OTU_223, OTU_228, OTU_230, OTU_234, OTU_235, OTU_236, OTU_239, OTU_24, OTU_240, OTU_241, OTU_244, OTU_245, OTU_246, OTU_249, OTU_25, OTU_251, OTU_252, OTU_254, OTU_256, OTU_257, OTU_259, OTU_26, OTU_261, OTU_262, OTU_269, OTU_27, OTU_270, OTU_274, OTU_275, OTU_276, OTU_28, OTU_281, OTU_283, OTU_287, OTU_29, OTU_290, OTU_292, OTU_296, OTU_298, OTU_30, OTU_301, OTU_303, OTU_309, OTU_31, OTU_310, OTU_311, OTU_312, OTU_322, OTU_323, OTU_324, OTU_328, OTU_33, OTU_330, OTU_331, OTU_333, OTU_336, OTU_34, OTU_342, OTU_345, OTU_346, OTU_348, OTU_35, OTU_355, OTU_358, OTU_36, OTU_360, OTU_361, OTU_362, OTU_363, OTU_364, OTU_37, OTU_373, OTU_374, OTU_375, OTU_379, OTU_38, OTU_382, OTU_383, OTU_384, OTU_385, OTU_386, OTU_395, OTU_398, OTU_399, OTU_400, OTU_41, OTU_412, OTU_413, OTU_415, OTU_416, OTU_417, OTU_418, OTU_420, OTU_421, OTU_422, OTU_424, OTU_426, OTU_433, OTU_435, OTU_438, OTU_439, OTU_44, OTU_441, OTU_442, OTU_443, OTU_445, OTU_447, OTU_448, OTU_449, OTU_450, OTU_451, OTU_453, OTU_455, OTU_457, OTU_459, OTU_46, OTU_461, OTU_462, OTU_463, OTU_467, OTU_472, OTU_473, OTU_474, OTU_477, OTU_48, OTU_482, OTU_483, OTU_484, OTU_485, OTU_486, OTU_488, OTU_491, OTU_492, OTU_493, OTU_494, OTU_495, OTU_497, OTU_498, OTU_499, OTU_50, OTU_506, OTU_508, OTU_51, OTU_510, OTU_513, OTU_514, OTU_52, OTU_520, OTU_523, OTU_524, OTU_525, OTU_526, OTU_527, OTU_528, OTU_529, OTU_53, OTU_532, OTU_533, OTU_534, OTU_535, OTU_536, OTU_537, OTU_538, OTU_539, OTU_541, OTU_544, OTU_548, OTU_549, OTU_552, OTU_555, OTU_557, OTU_558, OTU_559, OTU_56, OTU_561, OTU_565, OTU_57, OTU_571, OTU_581, OTU_583, OTU_584, OTU_586, OTU_59, OTU_595, OTU_603, OTU_606, OTU_608, OTU_609, OTU_61, OTU_610, OTU_612, OTU_614, OTU_615, OTU_616, OTU_618, OTU_621, OTU_622, OTU_624, OTU_625, OTU_626, OTU_627, OTU_63, OTU_630, OTU_631, OTU_632, OTU_636, OTU_637, OTU_640, OTU_642, OTU_645, OTU_646, OTU_647, OTU_649, OTU_65, OTU_650, OTU_655, OTU_656, OTU_657, OTU_659, OTU_66, OTU_660, OTU_664, OTU_666, OTU_667, OTU_668, OTU_669, OTU_67, OTU_670, OTU_671, OTU_674, OTU_68, OTU_680, OTU_684, OTU_685, OTU_687, OTU_688, OTU_689, OTU_690, OTU_692, OTU_694, OTU_695, OTU_696, OTU_699, OTU_70, OTU_701, OTU_702, OTU_705, OTU_71, OTU_713, OTU_715, OTU_716, OTU_718, OTU_721, OTU_722, OTU_726, OTU_73, OTU_733, OTU_737, OTU_738, OTU_739, OTU_740, OTU_746, OTU_751, OTU_752, OTU_753, OTU_754, OTU_76, OTU_761, OTU_765, OTU_768, OTU_771, OTU_772, OTU_778, OTU_779, OTU_78, OTU_781, OTU_783, OTU_784, OTU_785, OTU_789, OTU_79, OTU_790, OTU_792, OTU_793, OTU_796, OTU_797, OTU_80, OTU_800, OTU_803, OTU_804, OTU_807, OTU_809, OTU_81, OTU_810, OTU_812, OTU_813, OTU_82, OTU_823, OTU_83, OTU_831, OTU_832, OTU_833, OTU_84, OTU_843, OTU_847, OTU_849, OTU_851, OTU_853, OTU_857, OTU_859, OTU_86, OTU_862, OTU_864, OTU_866, OTU_868, OTU_87, OTU_872, OTU_875, OTU_876, OTU_88, OTU_889, OTU_89, OTU_891, OTU_894, OTU_895, OTU_896, OTU_897, OTU_898, OTU_900, OTU_901, OTU_903, OTU_908, OTU_909, OTU_913, OTU_914, OTU_915, OTU_916, OTU_92, OTU_922, OTU_925, OTU_928, OTU_929, OTU_930, OTU_931, OTU_932, OTU_935, OTU_936, OTU_937, OTU_938, OTU_939, OTU_94, OTU_940, OTU_941, OTU_943, OTU_944, OTU_948, OTU_949, OTU_953, OTU_954, OTU_958, OTU_96, OTU_963, OTU_965, OTU_969, OTU_97, OTU_971, OTU_972, OTU_976, OTU_977, OTU_979, OTU_98, OTU_986, OTU_987, OTU_988, OTU_99, OTU_990, OTU_992, OTU_993, OTU_997. |

**SUPPLEMENTARY TABLE** **4** Kruskal-Wallis test results of carbon sequestration phylum level under different slopes and different nitrogen additions

| Carbon-fixing bacteria phylum | *P*-value | | |
| --- | --- | --- | --- |
|  | G (nitrogen addition) | S (nitrogen addition) | G-S |
| Cyanobacteria | 0.15 | 0.80 | 0.04 |
| Actinobacteria | 0.54 | 0.28 | 0.05 |
| Proteobacteria | 0.10 | 0.54 | 0.13 |
| Ascomycota | — | 0.54 | 0.15 |
| Basidiomycota | — | 0.39 | 0.32 |
| Mucoromycota | — | 0.39 | 0.32 |
| unidentified | 0.39 | 0.28 | 0.60 |
| Streptophyta | 0.27 | 0.39 | 0.69 |

**SUPPLEMENTARY TABLE** **5** Carbon-fixing bacteria genera with significant differences in relative abundance of nitrogen addition at different slopes (relative abundance > 0.1%)

| Slope | Carbon-fixing bacteria genus | Relative abundance (%) | | | |
| --- | --- | --- | --- | --- | --- |
|  |  | CK | LN | MN | HN |
| Gentle slope | *Rhodovulum* | 2.25±1.80 | 7.56±6.60 | 6.34±3.08 | 19.66±12.01 |
| Steep slope | *Ectothiorhodospira* | 0.62±0.89 | 4.65±6.69 | 10.94±15.21 | 11.58±9.70 |
|  | *Cupriavidus* | 0.45±0.19 | 2.75±4.95 | 1.63±1.07 | 23.39±21.65 |

**SUPPLEMENTARY TABLE** **6** Multiple comparison of single factor AVOVA of Kruskal-wallis with different slope nitrogen addition treatments

| Slope | Carbon-fixing bacteria genus | *P*-value | | | | | |
| --- | --- | --- | --- | --- | --- | --- | --- |
|  |  | CK/LN | CK/MN | CK/HN | LN/MN | LN/HN | MN/HN |
| Gentle slope | *Rhodovulum* | 0.158 | 0.137 | 0.002 | 0.941 | 0.102 | 0.119 |
| Steep slope | *Ectothiorhodospira* | 0.299 | 0.017 | 0.012 | 0.181 | 0.137 | 0.882 |
|  | *Cupriavidus* | 0.882 | 0.181 | 0.007 | 0.234 | 0.012 | 0.181 |

**SUPPLEMENTARY TABLE** **7** Relative abundance of carbon-fixing bacteria in different slopes and Kruskal-wallis test results

| Carbon-fixing bacteria genus | Relative abundance (%) | | *P*-value |
| --- | --- | --- | --- |
|  | Gentle slope | Steep slope |  |
| *Cupriavidus* | 1.33±2.21 | 6.76±14.02 | 0.035 |
| *Alkalispirillum* | 1.22±2.54 | 8.02±8.25 | 0.000 |
| *Rhodovulum* | 8.88±9.17 | 2.50±6.35 | 0.001 |

**SUPPLEMENTARY TABLE** **8** Results of independent sample t test on soil physical and chemical properties, microbial biomass, carbon-fixing microbial community structure and diversity of degraded alpine meadow with different slopes in natural conditions

| Index | Variable | *F*-value | *P*-value |
| --- | --- | --- | --- |
| Soil physicochemical properties and microbial biomass | SOM | 0.054 | 0.340 |
|  | TN | 0.164 | 0.844 |
|  | TP | 0.096 | 0.740 |
|  | TK | 0.319 | 0.000 |
|  | AN | 0.149 | 0.564 |
|  | N-NO_3_^-^ | 0.971 | 0.387 |
|  | N-NH_4_^+^ | 4.298 | 0.633 |
|  | AP | 1.430 | 0.441 |
|  | AK | 14.931 | 0.606 |
|  | MBC | 0.002 | 0.020 |
|  | MBN | 1.500 | 0.553 |
|  | MBP | 2.791 | 0.257 |
|  | SWC | 6.410 | 0.852 |
|  | pH | 3.435 | 0.106 |
| Stoichiometric proportion | Soil C:N | 5.671 | 0.017 |
|  | Soil C:P | 0.004 | 0.286 |
|  | Soil N:P | 0.375 | 0.982 |
|  | Microbial C:N | 0.899 | 0.063 |
|  | Microbial C:P | 4.548 | 0.391 |
|  | Microbial N:P | 0.446 | 0.475 |
| Carbon-fixing bacteria phylum | Ascomycota | 9.000 | 0.356 |
|  | Cyanobacteria | 7.896 | 0.056 |
|  | Actinobacteria | 8.889 | 0.323 |
|  | Basidiomycota | 9.000 | 0.356 |
|  | Mucoromycota | 9.000 | 0.356 |
|  | Proteobacteria | 8.851 | 0.290 |
|  | Streptophyta | 28.815 | 0.186 |
| Carbon-fixing bacteria richness and diversity | Chao1 | 0.672 | 0.615 |
|  | Shannon | 0.027 | 0.977 |

Note: *P* < 0.05 in the table indicates that there is a significant difference in this index between gentle slopes and steep slopes.
